# The Role of a Succinyl Fluorescein-Succinic Anhydride Grafted Atactic Polypropylene on the Dynamic Mechanical Properties of Polypropylene/Polyamide-6 Blends at the Polypropylene Glass Transition

**DOI:** 10.3390/polym12061216

**Published:** 2020-05-27

**Authors:** Jesús-María García-Martínez, Emilia P. Collar

**Affiliations:** Polymer Engineering Group (GIP), Polymer Science and Technology Institute (ICTP), Spanish National Research Council (CSIC), C/Juan de la Cierva, 3, 28006 Madrid, Spain

**Keywords:** PA6/iPP, blends, glass transition, interfacial agents, compatibilizers, aPP-SFSA, DMA, wastes

## Abstract

The present article adequately supports a twofold objective. On one hand, the study of the dynamic mechanical behavior of polypropylene/polyamide-6 blends modified by a novel compatibilizer was the objective. This was previously obtained by chemical modification of an atactic polypropylene polymerization waste. On the other hand, the accurate predictions of these properties in the experimental space scanned was the objective. As a novelty, this compatibilizer contains grafts rather than just maleated ones. Therefore, it consists precisely of an atactic polymer containing succinic anhydride (SA) bridges and both backbone and terminal grafted succinyl-fluorescein groups (SFSA) attached to the atactic backbone (aPP-SFSA). Therefore, it contains 6.2% of total grafting (2.5% as SA and 3.7% as SF), which is equivalent to 6.2 × 10^−4^ g·mol^−1^. This interfacial agent was uniquely designed and obtained by the authors themselves. Essentially, this article focuses on how the beneficial effect of both PA6 and aPP-SFSA varies the elastic (*E*’) and the viscous (*E*’’) behavior of the iPP/aPP-SFSA/PA6 blend at the iPP glass transition. Thus, we accurately measured the Dynamic Mechanical Analysis (DMA) parameters (*E*’, *E*’’) at this specific point considering it represents an extremely unfavorable scenario for the interfacial modifier due to mobility restrictions. Hence, this evidences the real interfacial modifications caused by aPP-SFSA to the iPP/PA6 system. Even more, and since each of the necessary components in the blend typically interacts with one another, we employed a Box–Wilson experimental design by its marked resemblance to the “agent-based models”. In this manner, we obtained complex algorithms accurately forecasting the dynamic mechanical behavior of the blends for all the composition range of the iPP/aPP-SFSA/PA6 system at the glass transition of iPP.

## 1. Introduction

By considering both industrial and academic perspectives, it can be affirmed that the polyolefin/polyamide blend system remains a prime subject for years to come. This is thanks to its uses mainly related to the highly improved impact properties and much lower moisture absorption than neat PA6, becoming negligible once compatibilized [1,2,3,4,5,6,7,8,9,10,11,12,13,14]. Thus, this is one of the reasons why the authors, based on their own experience, used the iPP/PA6 system to evaluate the effect of this novel aPP-SFSA interfacial agent over dynamic mechanical behavior [10,11,12,13,14,15,16]. It is effectively worth indicating that, before studying the iPP/PA6 blend by including nearly all the complete compositional range, a series of previous basic concepts must be assumed. Thus, the previous knowledge about the improvement of thermal and mechanical properties, the evidence of the real reactive compatibilization occurring between the iPP and the PA6 phases, and the stability of the emerging morphologies even after post-processing steps must be taken into account [10,11,12,13,14,15,16].

The employment of compatibilizers, also named interfacial agents, with grafted groups onto a polymer backbone is a well-known and efficient method of improving the interactions between the components of the incompatible phases (here iPP and PA6). Therefore, the compatibilization of this type of polymer blends appears as a splendid example [1,2,3,4,5,6,7,8,9,10,11,12,13,14,15,16]. It should be mentioned that that this interfacial agent must be chemically similar to one of the phases in the blend. Besides, it must enhance its affinity to the other polymeric phase. According to this, it becomes evident that the use of novel interfacial agents becomes a choice for the understanding of the interfacial interactions, as well as to maximize the ultimate properties of whatever compound [14,15,16,17,18,19].

It needs to be mentioned that these two immiscible phases (iPP, PA6) must require enhancing the interactions at an interfacial level through a compatibilizer. And this one must resemble one of the phases with emphasis to the more mobile (iPP) [14,15,16,17,18,19], looking for an easy hosting of the interfacial agent. In addition, it must take in mind that the functionalities are mandatorily excluded from the crystalline domains [10,11,12,13]. As a result, this fact helps the interaction with the PA6 phase through real chemical bonds [10,14,15,16,17,18,19]. In this way, the better the interactions provoked by the interfacial agent, the better the properties obtained for the blends [10,14,15,16]. It is also noteworthy to mention that the interfacial agent used here (aPP-SFSA) has an amorphous origin [15,16,19]. This fact becomes an advantage for a better hosting into the amorphous phase present in the semi-crystalline iPP, allocating also the PA6 phase [14,15,16,19]. Moreover, to mention that being both phase (iPP; PA6) not rigid means that the inter-phase between them reflects a mobile character. In this way, the interactions between the amide groups present in the polyamide and the succinyl-fluorescein and succinic anhydride groups grafted in the interfacial agent are favored [11,12,13,14,15,16,19].

**Figure 1:**
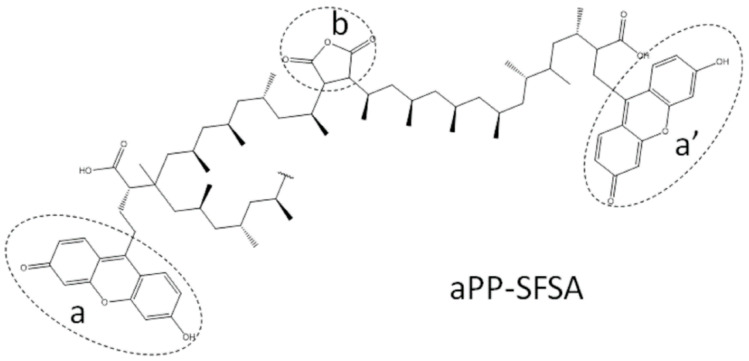
The chemical structure of the compatibilizer used in this work. (**a**) Side and (**a’**) terminal succinyl-fluorescein grafts. (**b**) Bridge succinic anhydride grafts.

To summarize, it is worth reminding that one of the purposes of this work lies in the investigation of the interfacial modifications by a compatibilizer obtained from polymerization wastes [20,21], and its use in a system wherein the interphase becomes mobile [14,15,16] rather than in particulate composite material wherein one of the phases is rigid (the reinforcement) [22,23,24,25,26,27,28]. It is worth mentioning that most studies in the literature always study PA6 contents lower than 50%, representing the rigid dispersed phase [3,9,10,11]. Thus, one novelty of this work lies in considering almost all the composition range (from 0.5% up to 99.5% in iPP) in the iPP/PA6 blend [10,12,13,14,15,16]. Equally, the use of this novel interfacial agent containing diverse types of grafts rather than just the maleated represents our original approach. Hence, this compatibilizer includes many types of grafts: succinic bridges between the polyolefin chains and succinyl-fluorescein grafted groups (both as terminal and backbone grafts) as plotted in Figure 1 [10,14,15,16,18,19]. As a novelty respecting our previous studies, this paper tries to demonstrate the real interfacial effect of aPP-SFSA even in the very constrained scenario (the glass transition of iPP) for the interfacial agent mobility. Conceivably to mention here that the glass transition is an interval wherein the iPP relaxation consists of short-range diffusive motions at the chain segment level [15,16,19,22,23,24,25,26,27,28,29,30]. Thereinafter, to indicate that most works in the literature concern to grafted interfacial agents based on isotactic polypropylene with maleic anhydride grafts (succinic once grafted) [19,22,23]. Accordingly, the use of an interfacial agent (of amorphous nature) is an innovative approach performed by the authors [10,11,12,13,15,16,23,25,26,27,28,29,30].

Another key purpose of the article is the prediction of the dynamic mechanical performance of the iPP/PA6 compounds of the iPP glass transition because it remains a critical point since a final performance perspective. The latter for whatever the composition range is chosen. Additionally, this allows exploring the control of the elastic/viscous balance behavior in the compound to a more precise understanding of the occurring interfacial phenomena. In this way, the Box–Wilson surface response methodology allows correlating the Dynamic Mechanical Analysis (DMA) parameters to the iPP/PA6 ratio and the amount of the interfacial modifier [31,32]. Additionally, just to remark that making Box–Wilson statistical design of experiments (sDOE) acquiring a physical sense (rather than merely empirical) implies to perceive what a complex system means. That is, a complex system (here the iPP/aPP-SFSA/PA6 system) must consist of many blocks (or items) exchanging stimuli between them and their surroundings. Equally, a complex system implies non-linear relationships. Consequently, the model utilized must be at least a quadratic one [33,34]. All the latter implies that the behavior of the system results extremely differently from the expected by merely considering the properties of the individual blocks in isolation (here iPP, PA6 and interface agent) [33,34]. The Box–Wilson response surface methodology may assume this idea since this model considers blocks (here named controlled factors) jointly to a series of interaction terms. The latter helps to detect other effects rather than merely external stimuli due to those of the blocks separately [33,34]. It is aptly worth mentioning that the processing operations are under equal confined flow conditions. Thus, some trace of external stimuli (if any) rather than of the iPP/interfacial agent/PA6 interactions results minimized in the model. Then, by considering all the above mentioned, this investigation implies the study and forecast of the storage (*E*’) and loss modulus (E”) at the glass transition of the more mobile phase (iPP). The latter determines the synergistic effect of iPP, PA6, and aPP-SFSA by identifying this critical concentration leading to improved properties.

## 2. Materials and Methods

### 2.1. Materials

For preparing the blends, we used an isotactic polypropylene, ISPLEN 050 (Repsol, Madrid, Spain), a Polyamide 6, Ultramid3 (BASF, Barcelona, Spain), and a waste origin grafted atactic polypropylene containing succinic bridge grafts jointly to the terminal and backbone succinyl-fluorescein grafts (aPP-SFSA) obtained by the authors [10,18,19,20,21].

The declared properties for iPP as received were: ρ = 0.90 g/cm^3^; *M*_w_ = 334,400 g/mol; *M*_n_ = 59,500 g/mol; *T*_m_ = 164 °C; and *T*_g_ = −13 °C. For the PA6 were: ρ = 1.13 g/cm^3^; *M*_w_ = 25,000 g/mol; *M*_n_ = 13,000 g/mol; *T*_m_ = 221 °C and *T*_g_ = 65 °C. And finally, the interfacial agent used contains 6.2% of total grafted groups, 2.5% as SA and 3.7% as SF (equivalent to 6.2 × 10^−4^ g/mol as succinic equivalent) [10,18,19,20,21]. The fully synthesis and characterization procedures of this interfacial agent were obtained by the authors and described elsewhere [10,18,19]. Figure 1 shows the chemical structure of aPP-SFSA.

### 2.2. Sample Preparation

Previously to the compounding, the PA6 pellets were conditioned for 48 h at 60 °C in order to remove the absorbed water. After what, the components (iPP, PA6, and aPP-SFSA) were dry mixed and dosed by following the Box–Wilson worksheet in Table 1 by considering that the interfacial agent replaces part of the iPP. Thereafter, the blends were compounded in a Rheomix 600 chamber connected to a Rheocord 90 by Haake (Barcelona, Spain) at a set temperature of 240 °C and 45 rpm of mixing rate during 5 min once the torque was constant. The blend obtained was rapidly cooled in a sudden by immersion into ice water. So, the material obtained was pelletized, and then kept in a vacuum oven for at least 12 h, and so molded. Thus, 3 g of material was compression molded under confined flow conditions by using a Dr Collin press (Barcelona, Spain) for 4 min at 270 °C by ensuring complete melting, and then one additional minute at 10 MPa pressure and 270 °C. After that, the mold was passed to a cooling cartridge applying a pressure of 20 MPa (packaging step) until reaching room temperature.

Afterward, we obtained circular sheets (diameter equal to 20 cm 0.1 mm thick). So, and with the purpose of avoiding the preferential flow lines of the molding step, a series of rectangular prismatic specimens (20 × 4 × 0.1 mm) were cut off in the circumferential sense of the sheets. Prior to performing the DMA test, all these samples were conditioned for 12 h at room temperature and 50% of relative humidity (RH).

### 2.3. Characterization Procedures

The dynamic mechanical characterization was performed in a METTLER DMA861 analyzer (Madrid, Spain), under the tension mode and operating at a dynamic force of 0.2 N and a fixed frequency of 1 Hz for proper identification of interfacial effects [15,16,35]. Additionally, and to maintain the sample in the visco-elastic behavior range, an amplitude of 4 μm and a heating rate of 2 °C/min were used as working parameters. The temperature range varied between −60 °C and 60 °C. The rather low values for both the frequency and the applied displacement were fixed to avoid morphological changes caused by internal heat generation, as well as to not disturb the nonlinear responses. Hence, from the DMA spectra, the values for the elastic or storage modulus (*E*’) and the viscous or loss modulus (E”) measured at the glass transition temperature of the iPP phase can be obtained.

### 2.4. Mathematical Model

The statistical design of experiment methodology (sDOE) used in the present work is the so-called Box–Wilson surface response central composite design [31,32]. In essence, this sDOE methodology consists in a central rotary composite design with a series of (2^k^ + 2k + 1) experiments, plus (2 + k) central or replicated runs, and where k is the number of the variables chosen (in our case iPP and aPP-SFSA) [31,32]. Therefore, it can be said that the model consists in the so-called factorial design augmented with a star design with a series of additional replicated runs for the central point coded as is (0, 0). In consequence, in our case, the experimental data-sheet consists is a series of 13 experiments, nine different from each other plus four replicas. Table 1 shows the Box–Wilson experimental data-sheet wherein both the coded and the controlled factors have been included.

As it can be appreciated, if we want to cover almost the whole composition interval in the iPP/PA6 blend, we must consider the range between 15% and 85% in iPP (or the inverted for PA6). Moreover, and in the case of the interfacial modifier (aPP-SFSA), the range between 3% and 15% was considered. In this way, the model covers the range between 0.5% and 99.5% in PA6; and the interval between 0.5% and 17.5% of interfacial agent. These intervals are the consequence of the factorial character of each component coded as (−1, 1) in the Box–Wilson methodology. Further, this considers α = $\sqrt{2}$ as the coded variable factor for the star points of the model [31,32]. Hereinafter, by fitting any measured property (*E*’ and *E*’’ in our case), a quadratic polynomial predicting the property in all the experimental range scanned (if adequate correlated) can be obtained [31,32].

## 3. Results and Discussion

### 3.1. Basic Background and DMA Spectra

When a portion of the polymer matrix is replaced by whichever interfacial agent, the interaction through the interface between phases is enormously varied [10,11,12,13,14,15,16]. Thus, the existence of a critical interaction level caused jointly by both the amount and the type of the interface agent employed must emerge. Additionally, note that this critical interaction value is equally directly related to the processing history of the material [24,25,26,27,28]. The latter remains a prominent concern that is many times ignored in several seemingly fundamental studies that consider the processing and shaping steps as trivial. Thence, the critically significant effect of the emerging morphologies generated (retaining key importance in the ultimate properties of the material) is dismissed. At that point, it is a fundamental aspect keeping in mind some fundamentals about Dynamic Mechanical Analysis (DMA) to facilitate the understanding of the discussion to a broader audience. That is, the study of the elastic and viscoelastic behavior of any material under the influence of cyclic stress or strain conditions. In this sense, any stress provoked by a modulated force can be identified by a complex modulus (E*). This one can be separated into two components, one in-phase with the strain (named as storage or elastic modulus, *E*’) and one out-of-phase (named as loss or viscous modulus, *E*’’), being E* = (*E*’2 + *E*’’2)^1/2^. The ratio between the loss and the storage modulus is named the loss or damp factor (tan *δ* = *E*’’/*E*’). It can be affirmed that this technique, if adequately used, becomes an incredibly effective tool to perceive the general complex behavior of the polymer-based material as a whole. Notwithstanding, it is significant to remark that DMA encounters the prime difficulty concerning the proper understanding of the observed macroscopic parameter in terms of its microscopic origin [35].

**Figure 2:**
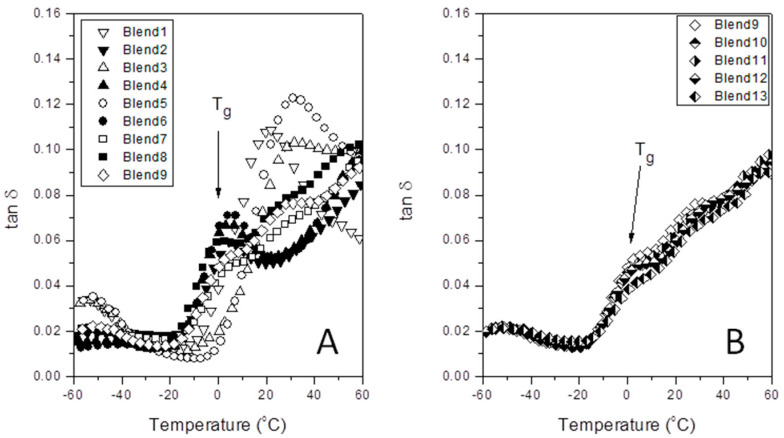
Loss factor (tan δ) versus temperature for the indicated samples. Determination of the glass transition of the iPP phase. (**A**) Central Rotary Composite Design Runs; (**B**) Central Point Replicated Runs.

The subject of this article represents the study of the DMA parameters for the iPP/PA6 system. And largely, it pays attention to how these ones change by the effect of a novel compatibilizer based on polymer wastes (aPP-SFSA) in an extremely unfavorable scenario (the glass transition of the polypropylene phase). Thus, Figure 2 shows the patterns of the damp factor (tan δ) versus temperature (T) for each experiment in Table 1. From the latter, we get the glass transition temperature of the iPP phase in the blend. This transition (*T*_g_) is typically located in the −10 °C and 40 °C intervals for polypropylene-based compounds [22,29,30]. Moreover, it is properly known that this is a transition related to the cooperative chain segments’ motion on the “free” amorphous phase of the polymer, wherein a short-range diffusive motion at the chain level takes place jointly to the significantly low stress (and so does heat) dissipation capability due to the mere atomic vibration motions [22,29,30]. We measured the glass transition from the tan δ peaks. Like so, we want to remark that the neat iPP obtains a value of *T*_g_ = 8.2 °C, as reported by the authors elsewhere [15,16,19]. Accordingly, it becomes evident that the presence of another phase (PA6) adjoined the interfacial agent (aPP-SFSA) must disturb the iPP phase, and, consequently, the glass transition temperature for iPP varies. Thus, to evaluate this aspect in a predictive sense, a Box–Wilson experimental model was implemented and employed in this work.

**Figure 3:**
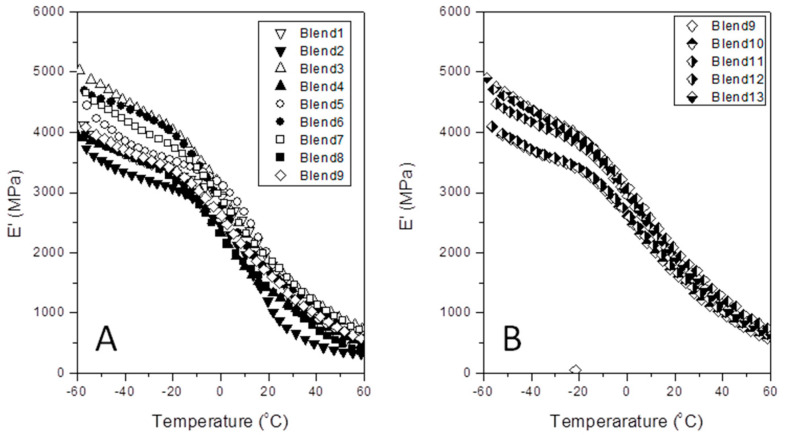
Storage modulus (*E*’) versus temperature for the indicated samples. (**A**) Central Rotary Composite Design Runs; (**B**) Central Point Replicated Runs.

**Figure 4:**
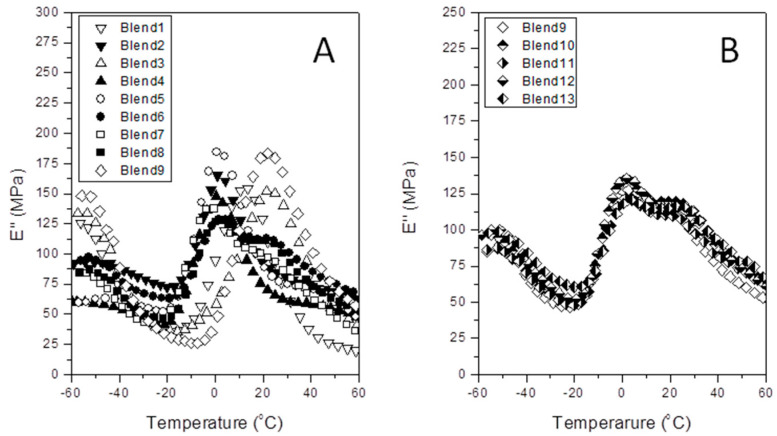
Loss modulus (*E*’’) versus temperature for the indicated samples. (**A**) Central Rotary Composite Design Runs; (**B**) Central Point Replicated Runs.

Otherwise, Figure 2, Figure 3 and Figure 4 represent the specific patterns of the various DMA parameters (tan *δ*; *E*’ and *E*’’) versus temperature for all the experiments satisfactorily performed. Here, we just used *E*’ and *E*’’ values as determined at the glass transition temperature from the tan *δ* plot. Table 2 lists the experimental worksheet, as well as the numerical values for *T*_g_, *E*’, and *E*’’ for each blend.

In our research, the values for *T*_g_ are between 4.8 °C and 0.0 °C. This notable difference of 4.8 °C may be provoked by the complex interactions of the iPP phase with the other components (PA6 and aPP-SFSA) in the blends. Notice that this difference increases up to 8.2 °C when compared to the *T*_g_ of the neat iPP as measured in the same way [15,16,19]. It is probably worth mentioning that the *T*_g_ of the neat iPP measured by DMA (*T*_g_ = 8.2 °C) differs remarkably from the one provided by the supplier (*T*_g_ = −13 °C) for two reasons. The first one is due to the alternative methods used by us (DMA versus DSC) [35,36]. The second is due to the profound effect of the mobility imprint caused by the confined flow conditions imposed by processing operations [15,16,19]. In this sense and as mentioned earlier, we wisely observe a maximum difference of 4.8 °C between the Blend 2 (iPP/aPP-SFSA ratio equal to 85.0/3.0; *T*_g_ = 4.8 °C) and the Blend 8 (iPP/aPP-SFSA ratio equal to 50.0/17.5; *T*_g_ = 0.0 °C). This is undoubtedly provoked by the combined effect of PA6 and the interfacial agent on the iPP glass transition, rather than the ones in isolation.

Accordingly, the authors tried to determine how the combined presence of the interface agent (aPP-SFSA) and PA6 appreciably affect the glass transition of the polyolefin phase, and then the storage (*E*’) and of the loss (*E*’’) modules at this transition, too. Consequently, Figure 3 and Figure 4 show the evolution curves of *E*’ and *E*’’ versus temperature for each one of the blends in Table 2. At a glance, we cannot properly distinguish a clear evolution pattern for these properties (*E*’ and *E*’’) as a function of the components of the bends. This aspect may be markedly linked to the complex interactions and the broad composition range intentionally chosen. A series of phenomena with antagonist effects may indeed be happening. Hence, adjoined with the well-accepted nucleation typically caused by the more rigid phase (PA6) on the iPP, the active role of the interfacial agent represents the apparent opposite. This is probably due to the remarkable fact that it comprises the amorphous phase of the polypropylene, which cohabits with both PA6 and aPP-SFSA. Consequently, this amorphous phase of iPP is much more constrained to freely participate in the motions related to the glass transition of iPP. Accordingly, the decay in this transition temperature is undoubtedly expected. Thus, when the iPP acts as the matrix, the amorphous interface agent (aPP-SFSA) may be homogeneously hosted in the iPP amorphous phase around the PA6 specific domains. Conversely, when PA6 is the matrix, the iPP exclusive domains face their amorphous phase to the same of PA6. At any rate, both the interfacial agent and the amorphous phases are excluded from the iPP crystals, obliging them to participate in the dissipation mechanisms intimately concerning their crystal/amorphous inter-phase [10,11,12,13,14,15,16]. Like so, we can typically observe distinct *E*’ values as a function of the amount of iPP (and so PA6) and the interfacial agent in the blend in all the specific temperature ranges studied. Namely, the more rigid domain (PA6) disrupts the polypropylene bulk, forcing a fraction of the macromolecular segments to be promptly ordered [14]. Consequently, the amorphous regions of iPP remain trapped at the iPP/PA6 interface. In other words, in the case that iPP acts as the polymer matrix, its amorphous phase is mainly coating the PA6 domains. However, when iPP represents the dispersed phase, this one surrounds the iPP domains facing the amorphous phase of the PA6 exclusive domains [10,11,12,13,14,15,16]. In any case, the amorphous phase of iPP must be highly constrained. Notwithstanding, this extremely complex scenario justifies the use of DOEs for a more proper understanding of the iPP/aPP-SFSA/PA6 system.

### 3.2. Polynomial Fits and Analysis of Variance (ANOVA)

The elastic (*E*’) and loss (*E*’’) modulus of all the blends precisely measured at the glass transition temperature of the polyolefin have been compiled in Table 2. Therein, the iPP amount (and indirectly PA6 content), and the interfacial agent (aPP-SFSA) remain the controlled factors. Thereafter, from *E*’ and *E*’’, quadratic polynomials modeling the elastic and the viscous behavior of the blend system were properly obtained by using the Box–Wilson surface response methodology [31]. Consequently, the terms of these, jointly to their <r^2^> and confidence factor coefficients (ANOVA), are compiled in Table 3. Accordingly, the <r^2^> values obtained were 84.1% for *E*’ and 86.6% for *E*’’, with both of them much higher than 75.0% and considered the threshold for a very good fitting for quadratic models [31,32]. Equally, the significantly high confidence factor values (98.3% for *E*’ and 98.8% for *E*’’) undoubtedly show the remarkable accuracy and high significance of the variables (iPP and aPP-SFSA) chosen by the model. In addition, Table 3 lists the “lack of fit” values. These are associated with the determined percentage of pure error related to possible factors overlooked by the model, but significant in the evolved response. Accordingly, all the specific parameters included in Table 3 state the likely possibility of diligently studying the blend behavior from the Box–Wilson model predictions.

**Figure 5:**
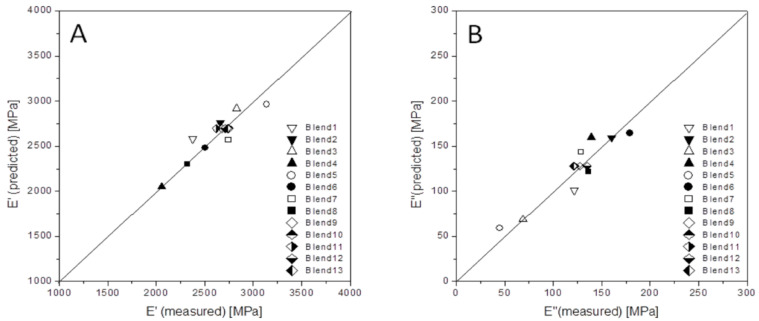
Measured versus predicted values for *E*’ (**A**) and *E*’’ (**B**) obtained at the iPP glass transition.

Notwithstanding, the model has to be mandatorily tested. Thus, in Figure 5, the predicted versus the measured properties for both *E*’ and *E*’’ are plotted, and a very good correlation between them is observed. Additionally, Table 4 compiles the *t*-values and the confidence coefficient (%) for the different terms of the polynomial obtained for the storage and loss modulus jointly with their significance levels for each one of the parameters [31]. So, since the prime influential parameters represent those with *t*-values higher than two, we observe that the effect of aPP-SFSA on *E*’ remains highly relevant (high values for both the linear and the quadratic term). This one is more aloft for the interaction term indeed, evidencing the complexity of the interactions occurring at the glass transition of the polyolefin phase of the blends.

By currently considering the viscous modulus (*E*’’), we can observe a different behavior. Here, iPP appears as the main responsible for the viscous behavior of the blends. Conversely, the interfacial agent fulfills a less meaningful role in this out-of-phase response. Put differently, this type of interfacial agent (aPP-SFSA) exerts further influence on the variation of the blends’ stiffness than on its loss response.

### 3.3. Influence of the Blend Composition in the Dynamic-Mechanical Behavior

Before adequately discussing the predictions of the model, we are obliged to point out a few key aspects of the experimental data compiled in Table 2. So, the fact that the uttermost considerable values of *E*’ and *E*’’ depend on the combined effect of both the interfacial agent and the polymer matrix remain evidenced. The higher compatibilizer amount does not lead to the extra change in the property (*E*’ or *E*’’) indicating that the iPP/aPP-SFSA/PA6 system gives rise to critical values for each component. This remark was previously detected by the authors elsewhere [11,12,13,14,15,16,17]. Notwithstanding, the subsequent sections discuss the evolution of both *E*’ and *E*’’ (measured at the glass transition of the iPP) by means of the model forecasts.

#### 3.3.1. Evolution of the Elastic Behavior

**Figure 6:**
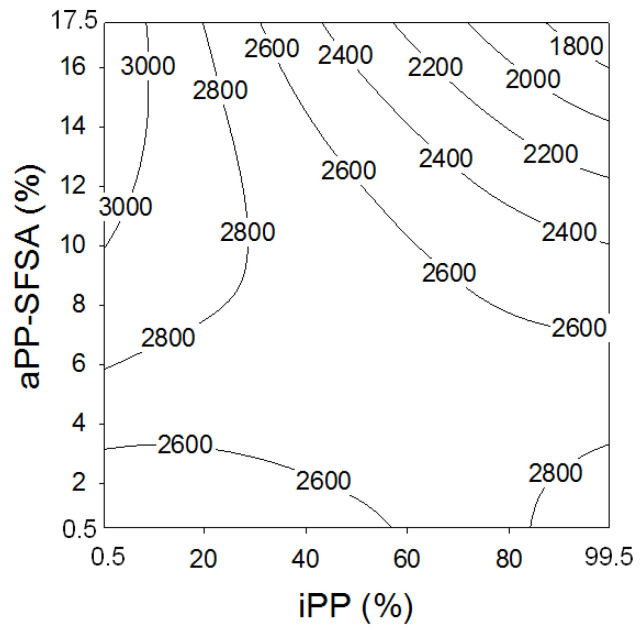
Contour plot of the storage modulus, *E*’ (MPa), as a function of the iPP and aPP-SFSA contents.

**Figure 7:**
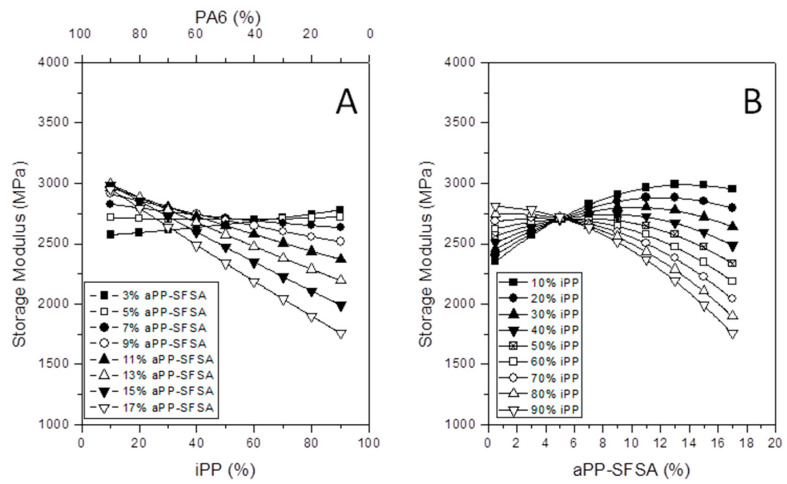
Evolution of *E*’ with iPP content for different amounts of interface agent (**A**). Evolution of *E*’ with the interfacial agent for different iPP contents (**B**).

The changes observed in the storage modulus evolution have been plotted in Figure 6 and Figure 7. Then, Figure 6 shows a contour map properly explaining the evolution of the storage modulus (*E*’) as a complex function of the iPP (and indirectly of the PA6 content) and the interfacial agent (aPP-SFSA). Besides, and due to the above-mentioned criteria about what the glass transition means (as an extremely unfavorable scenario) for the mobility of the aPP-SFSA used, some differences in *E*’ are found. Consequently, the active role of aPP-SFSA is convincingly demonstrated. Hence, Figure 6 shows the typical saddle or mini-max pattern contour plot for *E*’ for all the modified iPP/PA6 system, indicating the visible presence of critical coordinates in the experimental space scanned [31].

Namely, we typically observe that *E*’, for whichever the blend, prevail much higher significant than of the pristine iPP (*E*’ = 1567 MPa) [15,16,17]. At this place, we detect that just a scant amount of PA6 increases the modulus in more than 1000 MPa, mainly due to its nucleating effect on the iPP matrix [10,11,12,13,14,15,16]. However, we obtain significantly superior values for *E*’ (higher than 3000 MPa) in the specific case of the blend with nearly 90% in PA6 and aPP-SFSA higher than 10%. Nevertheless, the undoubtedly important revelation is that it is possible to obtain blends with almost the maximum stiffness (*E*’ = 2800 MPa) in all the compositional range just by varying the interfacial agent content. The above accurately indicates that the direct influence of iPP content on the *T*_g_ can be modulated by the active presence of aPP-SFSA. Moreover, the isolines in the contour plot are closer to each other for progressively increasing aPP-SFSA for iPP higher than 40% and aPP-SFSA higher than 8%. This evidences that high values for aPP-SFSA exercise a lower influence on the stiffness variations of the iPP/PA6 system. Therefore, the prime aspect found is the accurate identification of an optimum close to the phase inversion (50/50 iPP/PA6 ratio). Thus, this critical coordinate for *E*’ covers a broad interval between 35% and 85% in iPP content. And this stands notably influenced by the amount of aPP-SFSA, wherein there are throngs of possibilities to achieve the highest possible stiffness in the system by varying the amounts of the key components of the blend. Even more, we can also observe that, below around 40% iPP, just a little amount of aPP-SFSA is enough to provide remarkable beneficial properties. It is properly worth remarking that the use of Box–Wilson intentionally allows us to select the uttermost outstanding choice (in terms of concentration of the components) to obtain a previously stated desired performance. Consequently, these mean a great advantage if compared to conventional arbitrary experiments, wherein these critical points may be unidentified. Notwithstanding, these critical points were also pointed out in early works by authors intimately related to the thermal and mechanical properties at room temperature [12,13]. Hence, the above discussed properly indicates that aPP-SFSA is efficient in broad temperature and concentration ranges, as well as is coherent with the idea that aPP-SFSA (of amorphous character) may be better allocated in the amorphous phase of the iPP. Consequently, a considerable influence on the glass transition temperature is identified. Otherwise, this one was named “nonfree” amorphous phase by the authors, wherein the PA6 phase is also hosted (when it acts as the dispersed phase) [10,11,12,13,14]. In a similar fashion, when the dispersed phase is iPP, its amorphous “nonfree” phase remains faced with the PA6 amorphous phase typically surrounding the iPP domains [10,11,12,13,14].

By considering that the contour plots are not facile enough to be fully interpreted by throng potential readers, we also used parametric plots in Figure 7A,B. So, the presence of critical points is increasingly evident for any reader. Then, Figure 7A plots the storage modulus predictions, *E*’, versus the iPP and PA6 for different contents of aPP-SFSA. As follows, we observe that, for PA6 higher than 85% (15% iPP), the extra aPP-SFSA is the higher *E*’. However, close to the phase inversion (and even iPP higher than 40%), the effect obtains the reverse (the lesser interfacial agent the higher *E*’). The latter evidences the thoroughly efficient effect of aPP-SFSA in all the compositional range of the blend. And this aspect is significantly different from the effect of a similar interfacial agent, but simply maleated (aPP-SASA), published recently [16], indicating their completely different compatibilization mechanism. In the same sense, Figure 7B plots *E*’ versus aPP-SFSA. Here, two different pattern family curves depending on the critical content in aPP-SFSA can be observed. So, the behavior of whichever blend is the same when the content of the interfacial agent represents solely 5%. The latter supports the hypothesis that a significant amount of interfacial agent does not indicate a superior performance of the material as a whole. Moreover, below this coordinate, the higher PA6 content leads to improved properties. However, above 5% of aPP-SFSA, the behavior represents the contrary and much more crucial for *E*’. Therefore, we observe another critical point for aPP-SFSA content nearby 12%.

To conclude, just to remark that when PA6 is the dominating phase, the tendency of the curves is decreasing *E*’ with increasing aPP-SFSA when this one is higher than 5%, and the opposite happends when the amount of the other one is lower than this critical value. So, the existence of critical (or optimal) values for iPP, PA6, and aPP-SFSA in the blend maximizing the stiffness of the system is once more evidenced.

#### 3.3.2. Evolution of the Viscous Behavior

**Figure 8:**
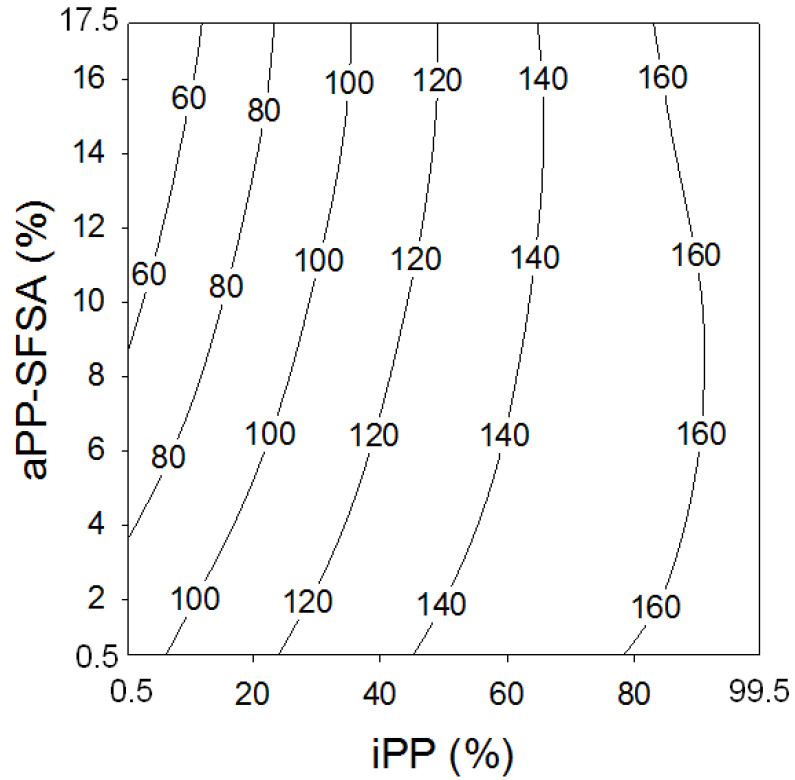
Contour plot of the loss modulus, *E*’’ (MPa), as a function of the iPP and aPP-SFSA contents.

**Figure 9:**
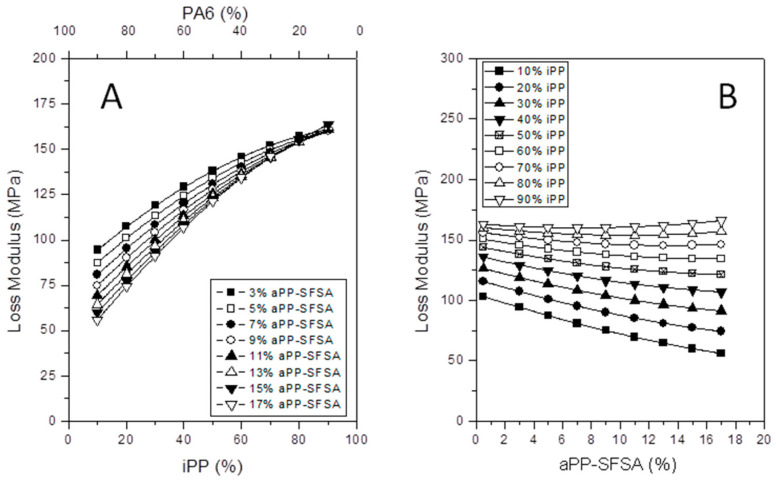
Evolution of *E*’’ with iPP content for different amounts of interface agent (**A**). Evolution of *E*’ with the interfacial agent at different iPP contents (**B**).

Figure 8 and Figure 9 plot the evolution of the loss modulus. So, Figure 8 shows the rising ridge pattern contour map, revealing the changes the loss modulus (*E*’’) underwent with the iPP and aPP-SFSA content. It is adequately worth mentioning that this specific kind of pattern naturally implies critical values of the controlled factors present in the whole investigated interval [31]. At this pivotal point, it is properly worthy to remark that, until very close to the inversion phase (50% iPP), the isolines follow a similar evolution and are remarkably close to each other. The intended above properly indicates a small influence of increasing amounts of the iPP phase in the viscous behavior of the whole iPP/PA6 system. However, we justly observe a beneficial effect of progressively increasing aPP-SFSA amounts for iPP lower than 60%. However, once surpassed, this 60/40 iPP/PA6 ratio, the viscous behavior (*E*’’) abruptly changes this complex pattern by exhibiting a critical point close to aPP-SFSA = 5%. Nevertheless, it is absolutely evident that, until iPP lower than 20%, *E*’’ through values are below the pristine iPP (*E*’’ = 95.5 MPa) [15,16]. This fundamental aspect remains chiefly remarkable for lower values of aPP-SFSA. The considered latter may indicate that the out-of-phase (viscous) behavior of the iPP/PA6 system (and very probably also the impact behavior) [36] is expected to increase for iPP higher than this threshold. It is important to remark here that, in this specific case, the iPP phase typically represents the dispersed phase. However, especially once surpassed the phase inversion (higher than 50%), increasing values for iPP implies more significant values for *E*’’. And so, the confirmed existence of a possible maximum (aPP-SFSA close to 5–6%) emerges naturally. This instantly appears to subtly suggest that a meaningful reorganization between the crystalline and amorphous phases of the iPP matrix has been undoubtedly taking place [10,14].

Accordingly, and for extra evidencing the critical points for this property, Figure 9A shows the evolution of *E*’’ as a function of iPP (and so PA6) and aPP-SFSA. Thus, the first remark to point out is properly the clear intercept point at 80% in iPP (20% PA6) as one of the critical coordinates of the blend system. Hence, it remains substantial that, below this key point, the higher aPP-SFSA the higher the loss modulus. This is much more increasingly evident with decreasing amounts of iPP (and increasing PA6). Here, the latter evidences the outstanding contribution of the interfacial agent to the viscous character of the system. That is, at the glass transition of the amorphous phase of iPP where it is allocated. Similarly, Figure 9B plots *E*’’ versus aPP-SFSA by varying the amount of iPP. Therein, we justly observe two families of curves. Therefore, we find one family of curves for iPP equal and/or lower than 50%. Here, the higher aPP-SFSA typically implies a gradual decrease in *E*’’ (although it implies a bountiful supply of other amorphous content to the blend). And, in this specific case, iPP remains higher than 50% with just the opposite evolution. Consequently, the more aPP-SFSA, the higher the out-of-phase modulus (*E*’’) is observed. The ones earnestly discussed over these lines adequately provide a straightforward idea about the extraordinary complexity of the reorganization phenomena typically occurring in the iPP/PA6 inter-phase. It is well worth mentioning that the latter governs the performance of the iPP/aPP-SFSA/PA6 system as a whole.

## 4. Conclusions

The potential effect of aPP-SFSA as an interfacial modifier in iPP/PA6 blends at the iPP glass transition was profitably studied. This was addressed by the combined use of the in-phase and out-of-phase behavior as obtained by DMA and further employment of statistical design of experiments (sDOEs). Moreover, aPP-SFSA revealed precisely being efficient for nearly every composition range of the iPP/PA6 system, even in the case of the very constrained scenario for the agent mobility that the glass transition implies. Additionally, the critical concentration coordinates for the components of the polymer blends are fairly evidenced. It is substantial to mention that, in this work, we obtained the same critical points as those predicted for the same blend by measuring other properties (mechanical, thermal, etc.) measured at very different temperatures. In any case, the Box–Wilson methodology revealed to be a very useful approach for diligently investigating a polymer-based heterogeneous material where the complex interaction between the components must naturally occur. Hereinafter, the genuine option of obtaining models able to predict the specific behavior for whatever possible iPP/PA6 blends is robustly evidenced, too. Hence, the above mentioned forecasting ability confirms that the design of materials with a previously selected set of properties becomes a real opportunity.

## Figures and Tables

**Table 1:** Worksheet of the Box–Wilson design used.

	Coded Factors		Controlled Factors	
Exp	iPP	aPP-*SFSA*	iPP (%)	aPP-*SFSA* (%)
Blend 1	−1	−1	15.0	3.0
Blend 2	1	−1	85.0	3.0
Blend 3	−1	1	15.0	15.0
Blend 4	1	1	85.0	15.0
Blend 5	−$\sqrt{2}$	0	0.5	9.0
Blend 6	$\sqrt{2}$	0	99.5	9.0
Blend 7	0	−$\sqrt{2}$	50.0	0.51
Blend 8	0	$\sqrt{2}$	50.0	17.49
Blend 9	0	0	50.0	9.0
Blend 10	0	0	50.0	9.0
Blend 11	0	0	50.0	9.0
Blend 12	0	0	50.0	9.0
Blend 13	0	0	50.0	9.0

**Table 2:** Storage and loss modulus at the indicated glass transition temperature of the iPP measured by the dump factor.

Exp	*T*_g_ (°C)	*E*’ (MPa)	*E*’’ (MPa)
Blend 1	4.0	2377.2	122.0
Blend 2	4.8	2658.0	160.0
Blend 3	2.8	2827.1	68.6
Blend 4	4.5	2058.7	139.0
Blend 5	0.4	3137.3	44.7
Blend 6	4.6	2507.2	178.9
Blend 7	3.4	2745.0	128.7
Blend 8	0.0	2319.1	136.2
Blend 9	2.6	2673.1	127.2
Blend 10	2.9	2748.2	135.0
Blend 11	3.0	2615.0	121.9
Blend 12	2.7	2707.0	135.5
Blend 13	3.0	2736.3	120.4

**Table 3:** Statistical parameters and coefficients of the polynomials from the Box–Wilson experimental design used. (Polynomial Equation: *a*_0_ + *a*_1_·*x*_1_ + *a*_2_·*x*_2_ + *a*_3_·*x*_1_·*x*_2_ + *a*_4_·*x*_1_^2^ + *a*_5_·*x*_2_^2^) *.

	<r^2^> (%)	Lack of Fit	Confidence Factor	Linear Terms			Interaction Term	Quadratic Terms	
		(%)	(%)	*a*_0_	*a*_1_	*a*_2_	*a*_3_	*a*_4_	*a*_5_
*E*’[MPa]	84.1	3.0	98.3	2253.4	5.21	112.5	1.25	0.01114	3.6490
*E*’’[MPa]	86.6	3.6	98.8	92.96	1.369	−4.429	0.03854	−0.00651	0.06522

* *x*_1_ = (iPP); *x*_2_ = (aPP-SF/SA).

**Table 4:** Confidence coefficient (%) and *t*-values for the different terms of the Box–Wilson model obtained for the *E*’ and *E*’’ moduli. (Polynomial Equation: *a*_0_ + *a*_1_·*x*_1_ + *a*_2_·*x*_2_ + *a*_3_·*x*_1_·*x*_2_ + *a*_4_·*x*_1_^2^ + *a*_5_·*x*_2_^2^) *.

	*Linear Parameters*		*Interaction Parameter*	*Quadratic Parameters*	
	x_1_	x_2_	x_1_·x_2_	x_1_^2^	x_2_^2^
*E*’[MPa]	0.96 (61.5%)	3.5 (98.9%)	3.7 (99.1%)	0.26 (26.0%)	2.5 (96.0%)
*E*’’[MPa]	2.12 (93.0%)	1.15 (70.0%)	0.98 (62.2%)	1.27 (74.5%)	0.37 (31.3%)

* *x*_1_ = (iPP); *x*_2_ = (aPP-SF/SA).
